# 35 m Vertical Free Fall: How Impact Surface Influences Survival

**DOI:** 10.1155/2014/805213

**Published:** 2014-10-23

**Authors:** C. Ehrnthaller, F. Gebhard

**Affiliations:** Department of Traumatology, Hand, Plastic, and Reconstructive Surgery, Center of Surgery, University of Ulm, Albert-Einstein-Allee 23, 89081 Ulm, Germany

## Abstract

We describe the accidental free fall of a 23-year-old construction worker, who fell 13 stories (approximately 35 meters) from a false work landing on a toilet container. On impact he broke through the roof of the container, which attenuated his fall and made his survival possible. The patient sustained a central spleen rupture, liver laceration, subdural hematoma, blunt thoracic trauma with a left-sided hematothorax and right-sided pneumothorax with serial bilateral rib fractures, and an unstable fracture of the 10th thoracic vertebra. Two thoracic drainages were inserted in the emergency department before the patient underwent emergency surgery for the management of his intra-abdominal injuries. On the third day after trauma the unstable fracture of the 10th thoracic vertebra was stabilized with an internal fixator. Following extubation on day 8 after trauma the patient did not show any peripheral neurological deficits but cerebral affection with a general slowdown. After only 21 days, the patient was discharged from the hospital to a rehabilitation center where work specific rehabilitation was started. Although the patient is not suffering from physical afflictions from the injury his daily life abilities are still limited due to cerebral damage.

## 1. Introduction

In most cases free fall injuries have high mortality rates with the majority of survivors suffering from a long-term morbidity [1]. With the critical border at a drop-height of about 30 meter, there are only several cases published for surviving an “unsurvivable” free fall injury.

In most published cases, body positioning on impact ameliorated the fall making survival possible [2, 3]. While several other factors are able to influence the injury severity as well (e.g., height, impact surface, and physical status of the patient [4–7]), there is no other case published to our knowledge where impact surface alone was able to attenuate a free fall combining an unsurvivable drop-height with an unfavorable body position leading to a fortunate clinical course with only mild physical long-term impairments.

## 2. Case Presentation

### 2.1. Medical History

**Figure 1:**
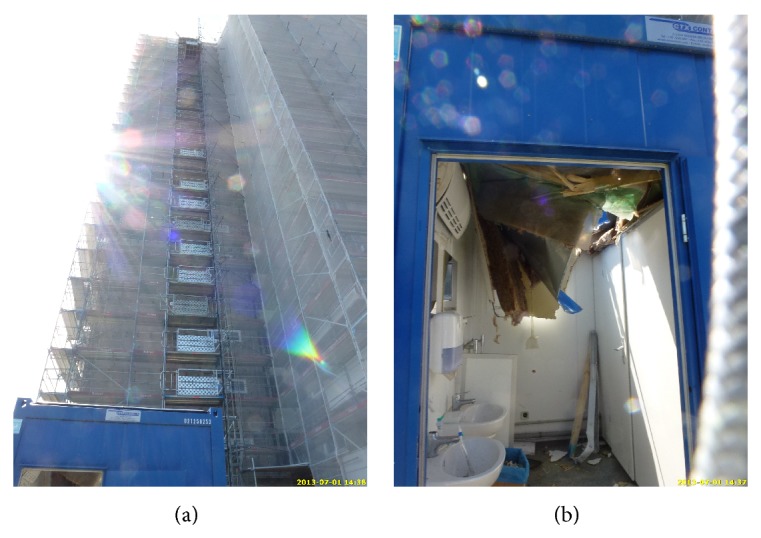
Pictures from the accident scene: (a) the scaffolding from which the patient fell is visible, (b) the destroyed rooftop of the toilet container after impact.

We report a 23-year-old patient who, due to unknown reason, fell from the 13th story of a scaffolding at an approximate height of 35 meters. Without hitting any other object, the patient fell onto the top of a toilet container, broke through the rooftop, and was found on the floor of the toilet container (Figures 1(a) and 1(b)).

When paramedics and the emergency physician arrived, the patient was gasping with a Glasgow coma scale of 3. After immediate intubation the patient did not generate sufficient oxygenation and was diagnosed with a left-sided tension pneumothorax. This was addressed by application of a (intravenous = IV) catheter into the left thorax at Monaldi position. After initial stabilization, the patient was admitted to our emergency department via ambulance.

### 2.2. Examination

On arrival to the emergency room, the patient was already intubated and ventilated; however he was hemodynamically unstable with a heart rate of 120 (beats per minute = bpm) and a hypotension (RR 85/45) (shock index 1.4). The clinical examination and immediate X-ray showed a bilateral unstable thorax with attenuated respiratory sounds. There were no signs of instability or injuries detectable on the upper extremities, the pelvis, or the lower extremities. Besides small bruising on the left knee and the left pelvis, there was a 5 cm long laceration of the galea occipitally. Upon arrival, the oxygen saturation was 88%, and initial blood gas analysis showed a hemoglobin count of 9.7 g/dL (other parameters: PaCO_2_ = 58; PaO_2_ = 59; Ca^2+^ = 1.14; BE = −9.7; lactate = 5.1). During further treatment the oxygen saturation fell to 84% with a hemoglobin of 7.8 g/dL.

### 2.3. Diagnostic Evaluation

**Figure 2:**
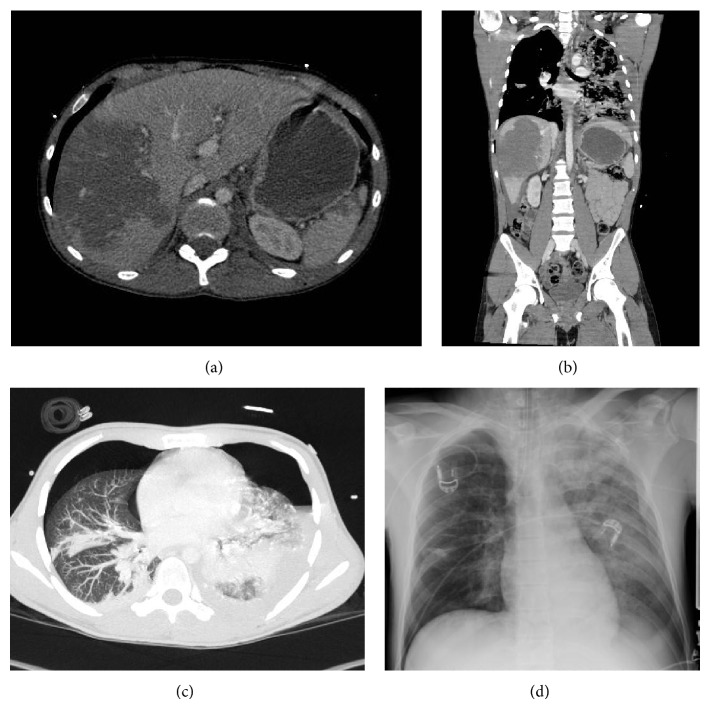
Radiologic evaluation of the intra-abdominal injuries: (a) and (b) imaging of the liver and spleen laceration with consecutive intra-abdominal hematoma in horizontal and frontal planes; (c) and (d) imaging of the hematothorax on the left side and pneumothorax on the right side in the horizontal plane and in conventional radiographs.

**Figure 3:**
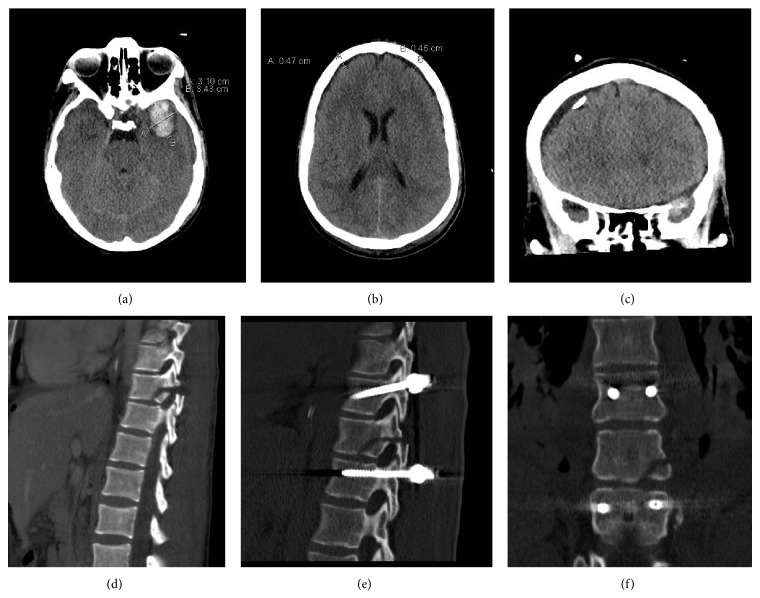
Radiologic evaluation of the intracranial and spinal injuries: (a) and (b) computertomographic imaging of the intracranial bleeding in the horizontal plane; (c) computertomographic control of the intracranial pressure probe; (d) sagittal imaging of the spine with unstable fracture of the 10th thoracic vertebra; (e) and (f) postoperative computertomographic control after dorsal instrumentation of the spinal fracture.

Immediate ultrasonography (FAST) showed free fluid in the left abdomen suspicious for a spleen laceration. After first stabilization, further diagnostic evaluation with computer tomography in the ER was performed. The following injuries were subsequently diagnosed:

(i) central spleen rupture with retroperitoneal hematoma (Figures 2(a) and 2(b));
(ii) blunt liver trauma grade III (central hematoma in the right lobe with several ruptures of the liver capsule in segments VI, VII, and VIII (Figures 2(a) and 2(b)));
(iii) subdural hematoma on the right temporal lobe (Figures 3(a) and 2(b));
(iv) hematothorax on the left side and pneumothorax on the right side (Figures 2(c) and 2(d));
(v) bilateral serial rib fractures (ribs 1–12 left side; ribs 1–5 and 9–11 right side);
(vi) fracture of the sternum with heart contusion;
(vii) fractures of the procc. transversi Th5-9 left side and Th10 bilaterally and procc. costalis L1 left side and L2/3 bilaterally;
(viii) multifragmentary fracture of the scapula with affection of the glenoid;
(ix) unstable fracture of the 10th thoracic vertebra with intraspinal dislocation of the posterior wall type AO B2 (chance fracture) (Figure 3(d)).

After review of all suffered injuries, the following trauma scores could be calculated:

(i) ISS: 66;
(ii) RISC: coefficient of −6.38 with a calculated mortality of 0.002 = 0.2%.

### 2.4. Initial Therapy

For stabilization of the coagulation cascade, 3 packs of fresh frozen plasma and 3 g of fibrinogen were administered in the emergency room. Thorax drainages were inserted in the Bülau position for the left-sided hematothorax and bilateral pneumothorax. At insertion the left-sided thorax drainage sucked 1 liter of blood out of the thorax. Consecutively, the heart rate increased from 100 to 120 and the blood pressure fell minimally from 135/60 to 120/60 with restoration after 5 minutes. After insertion, a conventional control X-ray was performed. However, due to the persistent hemodynamic instability and despite ongoing fluid resuscitation the patient was transferred to the operating theatre for surgical control of the intra-abdominal bleeding.

### 2.5. Further Clinical Course

In the surgical theatre a median laparotomy, splenectomy, and intra-abdominal packing of the liver laceration were performed. Intraoperatively, 10 packs of red blood cells, 9 packs of fresh frozen plasma, 4 g of fibrinogen, and 1 g of tranexamic acid were transfused. The abdomen was temporarily closed with a vacuum dressing. Parallel to the splenectomy, trepanation, and implantation of an intracerebral pressure probe (Figure 3(c)) a surgical wound closure of the occipital galea laceration was performed. Postoperatively, the patient was cardiopulmonally stable and was transported to the intensive care unit. Following the rules of damage control surgery, the unstable fracture of the thoracic spine was delayed until further stabilization of the patient [8]. Due to persistent volume requirements two more packs of fresh frozen plasma were transfused. Laboratory parameters returned to normal values in between the next 48 hours with decreasing lactate counts (5.1 at time of admission, 3.5 12 hours after trauma, and 1.0 24 hours after trauma) and increasing coagulation parameters reaching normal values on the second day after trauma. On the second day of trauma sedation of the patient was tapered and spontaneous movement of all 4 extremities was observed without any signs of neurologic impairment. On the third day after trauma, removal of the intra-abdominal packing in a second look operation was possible, where a cholecystectomy was performed due to ulcer formation of the gallbladder. Although a cerebral control computer tomography showed small new intracerebral bleeding and a slight increase of the cerebral edema, the dorsal stabilization of the spinal fracture was possible on the third day after trauma (Figures 3(e) and 3(f)). The spinal operation was performed in a minimally invasive technique (MIS) from Th9 to Th11 combined with an intraoperative navigation system (computer-assisted surgery = CAS) in a modern hybrid operating theatre [9]. On the fifth day, the secondary closure of the abdomen was performed.

Recurring reductions of sedative medication on the 6th and 7th day showed a delayed response and no adequate reaction of the patient. On the 8th day after trauma the patient was extubated successfully and on the 10th day after trauma the thorax drainages were removed. In the following course, the patient was mobilized and showed a general slowdown without any other signs of neurologic impairment and thus a diagnosis of diffuse axonal damage was suspected. In the following days the patient returned step by step to a normal diet and was gradually mobilized. Neurologic rehabilitation was organized and on the 21st day after trauma the patient was transferred to the rehabilitation center. During the stationary rehabilitation an intensive interdisciplinary therapy was performed. Besides main emphasis on mobilization techniques at the beginning and necessary analgetic treatment due to persistent pain in the area of the serial rib fractures in the further course ergotherapeutic, psychotherapeutic, and logopedic therapies became increasingly important. Overall, the rehabilitation showed a very positive course. With rehabilitation still ongoing, there are only minimal signs of physical impairment with a slightly decreased range of elevation of the left arm and intermediate episodes of sharp pain in the rib area. After nearly 8 months the patient is completely independent with ongoing impairments regarding emotional regulation, poor concentration, fine motor skills, and word finding disorders. However, despite initiation of scholar and job specific training the patient still requires assistance regarding complex mathematic exercises and has unrefined complicated working methods on occasion.

## 3. Discussion

In this case report the patient suffered from a 35 m free fall injury. Due to unknown reasons the patient fell approximately 13 stories and landed on a toilet container, breaking through the rooftop and landing on the container floor.

After initially necessary cardiopulmonary resuscitation and emergency hospital care the patient survived the fall and was able to be transferred to a rehabilitation center, after 21 days, to commence specific rehabilitation techniques.

The impact of the body onto the surface after a fall from great height leads to an acceleration of the body, which can vary depending on the height. The direct impact leads to transformation of the kinetic energy into deformation of the patient's body and the impact surface finally leading to tissue destruction in the patient's body [10, 11].

Based on properties like weight of the patient and fall height a free fall time of 2.67 seconds, leading to a velocity of 26.2 m/s and a kinetic energy of 24007 Joules on impact, could be calculated for our patient. Depending on the properties and stiffness of the impact surface, the kinetic forces leading to tissue destruction may be attenuated [12].

One major factor for survival of the patient is the surface of the impact. The rooftop of the toilet container is made of an initial layer of steel-paneling with a strength of 0.6 mm followed by a polyurethane-foam insulation (100 mm) and a chipboard with a strength of 10 mm. The rooftop is completed with a 10 mm drywall on the inside of the container (with additional stabilization being achieved by wooden beams). The overall size of the container is 2.98 meters in length, 2.43 meters in width, and 2.59 meters in height. The carrying capacity of the rooftop is described as 125 kg per m^2^.

Kurtz et al. published cases from 4 survivors jumping from a 50 m high bridge falling onto water. Compared to water, a free fall of the same height onto solid ground should have resulted in nearly 100% mortality [13]. With respect to the kinetics of the impact, several variables have to be respected. Basically, the duration of impact determines the force that is applied to objects. In addition, the duration of impact is dependent on the stiffness of both colliding objects, resulting in a minor force transformation to collision partners with low-compared to objects with high stiffness.

Every object can be characterized by its ability for elastic/plastic deformation and the timeframe in which deformation occurs. While concrete has a very high elastic modulus resulting in brittleness, water has a very low elastic modulus and is able to attenuate the impact by strong deformation of the surface and energy transformation over a relatively long period of time [14]. As the elastic modulus of a human body should be comparable between individuals the characteristics of the impact surface are more important for the outcome of the patient.

Bertocci et al. were able to demonstrate the alleviating role of the impact surface on the injury severity with significantly less head acceleration when falling onto playground foam compared to wood, linoleum, or padded carpet [15]. In the present case the impact was alleviated by falling onto the relative soft rooftop of the toilet container with a small elastic modulus (steel-panel, polyurethane-foam, and chipboard) resulting in a strong deformation of the impact surface and an energy transformation over a long period of time. The patient was lucky enough to hit the middle of the toilet container as an impact on the side-frame or the ground next to the container would have ended in a lethal outcome due to the robust metal beams on the side or the hard soil next to the container.

The most important prediction marker for mortality of free falls surely is drop-height. In a study with 180 free fall patients the mean height for nonsurvivors was 8.6 m ± 2.3 m and 5.2 m ± 0.2 m for survivors [16]. Another retrospective study demonstrated an average fall height of 7.2 m, additionally indicating the critical border for survivable injuries at a fall height of 6 to 7 m [1]. Sadly, both studies did not take into account the properties of the impact surface and they missed to include the patients dying at the scene or during the transport to the hospital. As it is generally known that the stiffness of the impact surface may attenuate the fall, the presented border for an unsurvivable drop-height should be interpreted with caution and may be underestimated regarding height due to the missing of the immediate deaths. In contrast to the aforementioned studies by inclusion of the out-of-hospital deaths Lapostolle et al. demonstrated 100% mortality in patients falling from 8 stories or greater (approximately 25–30 m) [17].

Another relevant parameter for survival of such accidents is the body position at the time of impact. While a free fall accident from the first floor onto the head shows a lethal outcome in almost 50% of the cases, if the body position is changed to a feet-forward position, then the prediction of survival is much higher [1, 14, 17]. Injury characteristics change with the position of a fall. A feet-forward position results in such accidents leading mainly to chain injuries of the lower extremity with fractures of the foot, ankle, and the long bones such as the tibia or femur [1, 14]. A sitting position at the time of impact more likely leads to injuries of the pelvis or spine. As already mentioned, the highest mortality is seen in headfirst accidents with the predominant injury distribution being traumatic brain injury with fractures of the cerebral or thoracic spine together with injuries of the shoulder girdle or thorax [14].

In a casebook of 100 free fall accidents the most important injury patterns were analyzed [1]. In this study spinal injuries were most frequent (83%) followed by lower extremity (44.6%) and injuries of the head (26.7%). These main injuries were followed by affection of the upper extremity (24.8%), thorax (20.8%), and pelvis (17.8%). In contrast, abdominal injuries only accounted for 5.9% of all cases [1]. Further examination of the spinal injuries reveals fractures of the 12th thoracic vertebra in 85.3% of all cases and in 52.9% these fractures are associated with neurologic symptoms [1]. The 10th thoracic vertebra, which was fractured in this case report, is only affected in 20% of all cases. But also the fracture morphology in this case is pretty unusual. Contrary to the blunt impact in this case a chance fracture most often happens through a flexion/distraction mechanism acting against a fulcrum [18]. But there are also several cases, where blunt trauma led to the development of a chance fracture [19, 20]. As in this case the fracture is located on the left dorsolateral side a blunt impact on the left dorsolateral side of the body is postulated. The crucial role of the body position was impressively demonstrated by a case report of a 90-meter free fall climbing accident. With the first impact at about 60 meters, the patient hit the ground feet-first and then fell onto pelvis and spine. Several authors called this the “ideal” body position because the kinetic energy of the impact is first absorbed by the nonessential parts of the body like the lower extremities before life-essential inner organs of abdomen and thorax are affected [2].

After careful analysis of the injury distribution pattern in this case with a generally more severe affection of the left side with severe lung contusion, scapula fracture, laceration of the occipital galea, dislocated serial rib fractures on the left side, and additional bilateral fractures of the procc. transversi, the patient most likely hit the toilet container with the left dorsolateral side first. Analyzing the morphology of rib fractures can tell a lot about their development. There is a wealth of knowledge that a direct impact onto the thorax leads to inwardly pointed rib fractures. In contrast, indirectly fractured ribs are pointed outwardly and occur after compression of the contralateral thorax [21]. The fact that fractured parts of the left ribs were pointed inwardly and the rib fractures of the right side did not show any significant dislocation strengthens the hypothesis of a dorsolateral impact. As this body position is linked with a substantial increase of mortality, the survival without any major physical impairment seems to be even more surprising [2, 4, 22, 23].

## 4. Conclusion

This case report clearly shows how external variables such as impact surface may affect injuries with a statistical mortality of 100% making survival possible. By solely contemplating height or body positioning of the free fall accident a lethal outcome should have been concluded.

The relatively low stiffness of the impact surface was able to convert the amount of kinetic energy acting on the patient's body compensating not only the great height but also the unfavorable body position of the patient at the time of impact. Besides a lucky survival the patient showed a surprising clinical course with discharge of a level 1 trauma center after only 21 days of treatment into a rehabilitation center with reversible neurological affections but missing significant musculoskeletal impairments. This unique clinical course was unsuspected and a definitive explanation is hardly possible.

On the other hand the clinical course also demonstrates the complexity of regaining all necessary skills for daily life, self-dependency, or even reintegration into the professional world with still ongoing, multidisciplinary rehabilitation.
